# Multi-Class Parrot Image Classification Including Subspecies with Similar Appearance

**DOI:** 10.3390/biology10111140

**Published:** 2021-11-05

**Authors:** Woohyuk Jang, Eui Chul Lee

**Affiliations:** 1 Department of Computer Science, Graduate School, Sangmyung University, Hongjimun 2-Gil 20, Jongno-Gu, Seoul 03016, Korea; woohyuk1204@naver.com; 2 Department of Human-Centered Artificial Intelligence, Sangmyung University, Hongjimun 2-Gil 20, Jongno-Gu, Seoul 03016, Korea

**Keywords:** object detection, deep neural network, parrot classification, CITES, illegal transaction

## Abstract

**Simple Summary:**

Owing to climate change and human overdevelopment, the number of endangered species has been increasing. To face this challenge, the CITES treaty has been adopted by many countries worldwide to prevent the extinction of endangered plants and animals. Additionally, since customs clearance inspections for goods at airports and ports take a long time, and due to the difficulty of distinguishing such species by nonexperts, smugglers have been exploiting this vulnerability to illegally import or export endangered parrot species. If these cases continue to increase, the extinction of species with fewer populations can be accelerated by illegal trade. To tackle this problem, in this study, we constructed an object detection model using convolutional neural networks (CNNs) to classify 11 endangered species of parrots. Utilizing artificial intelligence techniques, the procedures for inspection of goods can be simplified and the customs clearance inspection systems at airports and ports can be enhanced, thus protecting endangered species.

**Abstract:**

Owing to climate change and human indiscriminate development, the population of endangered species has been decreasing. To protect endangered species, many countries worldwide have adopted the CITES treaty to prevent the extinction of endangered plants and animals. Moreover, research has been conducted using diverse approaches, particularly deep learning-based animal and plant image recognition methods. In this paper, we propose an automated image classification method for 11 endangered parrot species included in CITES. The 11 species include subspecies that are very similar in appearance. Data images were collected from the Internet and built in cooperation with Seoul Grand Park Zoo to build an indigenous database. The dataset for deep learning training consisted of 70% training set, 15% validation set, and 15% test set. In addition, a data augmentation technique was applied to reduce the data collection limit and prevent overfitting. The performance of various backbone CNN architectures (i.e., VGGNet, ResNet, and DenseNet) were compared using the SSD model. The experiment derived the test set image performance for the training model, and the results show that the DenseNet18 had the best performance with an mAP of approximately 96.6% and an inference time of 0.38 s.

## 1. Introduction

Owing to climate change and human indiscriminate development, the number of endangered animal and plant species has been increasing. To tackle this problem, the Convention on International Trade in Endangered Species of Wild Fauna and Flora (CITES) has been adopted by many countries worldwide. It is an international agreement that requires approval for the import and export of registered animals and plants. Animals and plants designated by CITES are protected internationally.

Traditionally, the process of customs clearance inspection for goods at airports and ports has been done manually to check documents and real objects, making it a time-consuming and costly task. In particular, customs inspections for animals and plants can be inaccurate due to the difficulty of distinguishing species by nonexperts. Exploiting this vulnerability, smugglers illegally import or export animals and plants designated as endangered by CITES. Among them, parrot species are illegally traded [1]. If this trend continues, species with small populations may become extinct rapidly. Additionally, illegal wildlife trade is increasing every year [2], which has an adverse effect on biodiversity worldwide [3,4]. To tackle this problem, the automation of the customs clearance system for specific animal species based on artificial intelligence technologies and object detection methods can be a good tool. Object detection (i.e., the identification of a specific area in an image) is widely used in video monitoring [5,6,7], medical diagnosis [8], and many other fields.

In this study, we propose a method to classify 11 species of parrots designated as endangered by CITES using artificial intelligence techniques. The proposed method uses a convolutional neural network (CNN) to extract features from images and creates an object detection model for classification. The generated object detection model is an object detection model for classification of multiple parrot species, rather than simple object detection and image classification for a single species. To our knowledge, it is one of the few studies that have developed an artificial intelligence-based object detection model for species of parrots designated as endangered by CITES. The remainder of this paper is organized as follows. In Section 2, we review the literature on CNNs. Section 3 describes data acquisition and the preprocessing tasks and presents our model. The results of endangered parrot species classification are provided in Section 4. Additional explanations on the contributions and experimental results of this study are provided in Section 5. Finally, Section 6 summarizes the conclusions.

## 2. Related Work

### 2.1. CNN-Based Biological Image Recognition

CNNs have been a cornerstone in the field of computer vision since AlexNet [9], which won the ImageNet [10] classification competition in 2012. Since then, other deep CNN structures, such as VGGNet [11] and GoogleNet [12], have appeared. It has been shown that the classification quality can be greatly improved using low-level to high-level features through a deep structure. However, if you simply deepen the model structure, ‘degradation’ problems occur. To solve this problem, the ResNet [13] model was proposed.

The ResNet model solves the ‘degradation’ problem in deeper structures using skip connections that jump over some layers and add features that were used in the previous layer. Unlike ResNet, the DenseNet [14] structure connects directly to all network layers and reuses all features of the previous layer to maximize information delivery. Many studies have been conducted on biological image recognition and species classification using deep learning models with research being currently in progress.

Kang et al. proposed a deep learning model that can classify toxic and edible mushrooms so that the general public can distinguish between them [15]. Nguyen et al. and Norouzzadeh et al. developed a deep learning model for classifying animal species that can be used as an animal recognition system using camera traps [16,17]. Kim et al. presented a classification model for five species of wild animals found mainly in national parks [18]. In addition, an object recognition model was proposed for marine species classification without human intervention [19,20]. Further, Jang et al. developed a bird detection model for a single species using Faster R-CNN, with an average precision (AP) of 87% [21].

In this paper, we propose an object detection model for multiple species, rather than simple object detection and image classification for a single species.

### 2.2. Framework for Object Detection

The traditional method of object detection uses a support vector machine (SVM) [22] based on handcrafted features [23,24]. However, in recent years, the CNN model has been advancing, showing a superior performance. The first model to initially succeed in object detection by applying CNNs was R-CNN [25]. Since then, it has evolved into SPPNet [26], Fast R-CNN [27], and Faster R-CNN [28]. Since these models use a two-stage method that learns by separating regression and classification, the mean average precision (mAP) [29] is high, but the detection speed is low, making it difficult to be used for real-time applications. Recently, the one-stage method has become popular.

The one-stage approach is a method of learning classification and regression simultaneously. Representatively, the “You Only Look Once” (YOLO) [30] and Single Shot Multibox Detector (SSD) [31] models are examples of this method. Since classification and regression are simultaneously learned from the feature map generated through the convolutional layer, both the mAP and detection speed are high. Therefore, it is suitable for real-time applications.

The YOLO model conducts classification and regression using the last layer feature map of the backbone network and is measured at 45 frames per second (FPS) and 63.4% mAP. Further, the SSD model conducts classification and regression using multiple feature maps from the backbone network feature map and the convolutional layer and is measured at 59 FPS and 74.3% mAP. YOLO v3 [32] is based on the YOLO model, which uses the backbone network as Darknet-53 [32] and performs classification and regression using three feature maps of various size resolutions. The mAP performance of YOLO v3 is comparable to the SSD measured at 22 FPS. Lin et al. [33] used the RetinaNet model, which uses pyramid-shaped ResNet-FPN as the backbone and performs classification and regression using two subnetworks. In addition, low accuracy was derived due to object and background class imbalance in the one-stage method. To solve this problem, they proposed focal loss to replace cross entropy loss. Performance increased by 4% over SSD and YOLO models with similar speed results.

## 3. Materials and Methods

### 3.1. Dataset and Preprocessing

We constructed our own database to train the proposed SSD model. The dataset images were collected from the Internet and obtained in cooperation with Seoul Grand Park Zoo where an RGB camera was used to capture videos from various angles and extract video frames to create RGB images. Obtaining images at the zoo provided flexibility in terms of photo angles and poses; however, the image background was limited. Images collected from the Internet were also RGB images with various backgrounds for the same species; however, they were still image lacking flexibility of angles and poses. Therefore, the two sources were used for image collection to combine their advantages. For the preprocessing process, it is important to reduce the input image size to perform efficient calculations to construct the deep learning model.

**Figure 1:**
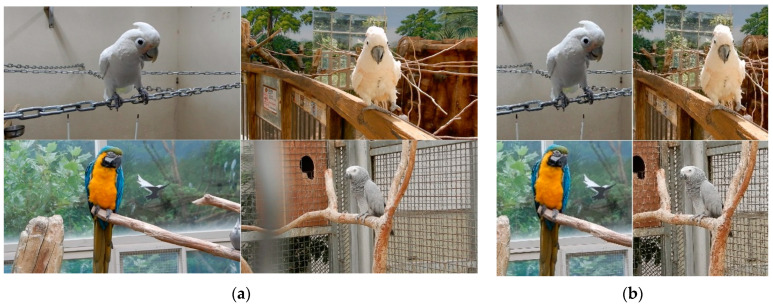
Examples of preprocessing. (**a**) before image resizing (1920 × 1080 pixel resolution), (**b**) after image resizing (300 × 300 pixel resolution).

Therefore, we cropped the extracted video frame to the object and then resized the image. Figure 1 shows the results of image resizing after cropping. This operation converted the original image with a resolution of 1920 × 1080 pixels into an area with a resolution of 300 × 300 pixels. In addition, since parrot species have different features for their eyes, beaks, and crests, we labeled them using features for the head area. Darklabel [34] tool was used for labeling.

**Figure 2:**
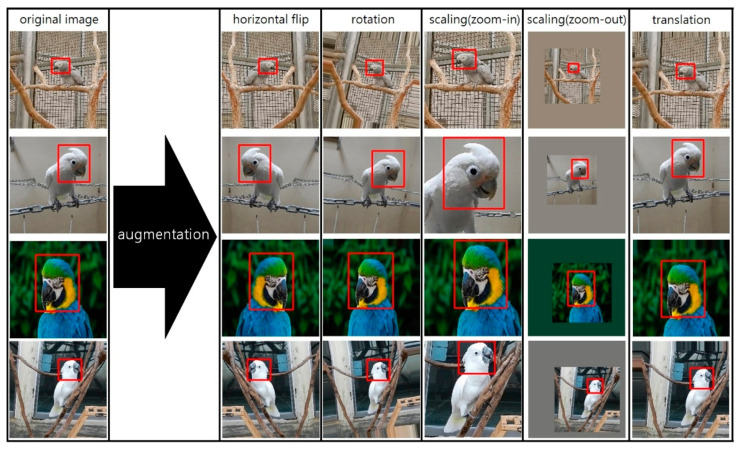
Data augmentation methods; red box represents the ground-truth.

We used data augmentation methods to limit data collection and prevent overfitting. Four techniques were applied: horizontal flip, rotation, scaling, and translation. Thus, we increased the dataset size and applied the parrot specific pose geometrically to prevent overfitting. The labeled image and data augmentation methods are shown in Figure 2. Our dataset consisted of 70% training set, 15% validation set, and 15% test set approximately, with 3040, 648, and 678, respectively. The data augmentation was applied only to the train set. Table 1 presents our dataset of 11 species of parrots to which data augmentation was applied, while Table 2 provides an overview of the 11 parrot species (scientific name: *Cacatua goffiniana*, *Cacatua ducorpsii*, *Cacatua alba*, *Cacatua moluccensis*, *Cacatua galerita*, *Ara macao*, *Ara ararauna*, *Camelot macaw*, *Ara chloropterus*, *Trichoglossus haematodus*, *psittacus erithacus*). Table 2 Appendix I refers to species that are or may be affected by international trade as endangered species. Their international trade is prohibited for commercial purposes and only allowed for academic research purposes. Table 2 Appendix II refers to species threatened with extinction if international trade is not strictly regulated. International trade for commercial, academic, and research purposes is possible, but restrictions are applied.

### 3.2. Deep Neural Networks

Deep learning is well known for being a powerful technique, not only in image classification, but also in regression problems. As mentioned in Section 2.2, several object detection models were introduced, but we compared the YOLO and SSD models among one-stage detection methods in this study. The SSD model outperforms the YOLO model.

**Figure 3:**
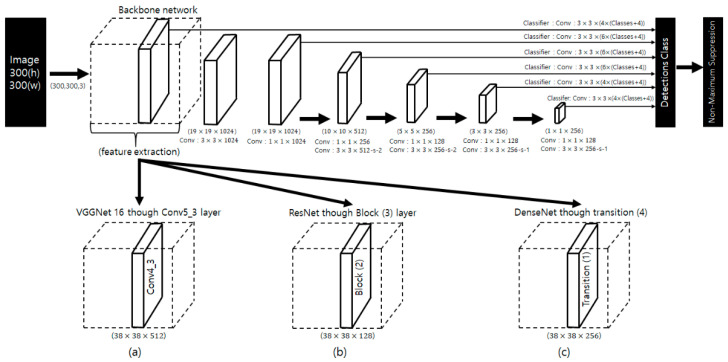
Parrot species classification using deep neural networks: (**a**) VGGNet, (**b**) ResNet, (**c**) DenseNet.

In this study, we developed a classification model for 11 endangered parrot species using the SSD model. Figure 3 shows an SSD model for the classification of the parrot species. The SSD model has an input image size of 300 × 300 pixels, and the predicted bounding box is identified using the feature map extracted from the backbone network and the feature map extracted using the bottleneck structure. In other words, it uses a multiscale feature map. As shown in Figure 3, the classifier (classification and regression) was applied using convolutional layer on a multiscale feature map. In addition, the SSD uses the anchor box concept of Faster R-CNN [28], which creates a default box by various scales and aspect ratios and maps it to a multiscale feature map to employ the classifier. With this operation, a feature map with high resolution detects small objects in the image, while a feature map with low resolution detects large objects. It also uses important information using low-level and high-level features. To train a model, one needs to choose an appropriate loss function that can perform multi-classification and the bounding box regression simultaneously. The loss function can affect the learning model. The loss function [31] used here is calculated as a linear combination of localization loss (loc) and confidence loss (conf) (see Equation (1)):

(1) $$L_{total}\left(x,c,\;l,\;g\right)=\frac{1}{N}\left(L_{conf}\left(x,\;c\right)+\alpha L_{loc}\left(x,\;l,\;g\right)\right),$$

where *N* is the number of default matched bounding boxes; x, c, l, and g represent image, class, predicted bboxes, and ground truth bboxes, respectively; and α is a value between 0 and 1.

(2) $$L_{loc}\left(x,\;l,\;g\right)=\overset{N}{\underset{i\in Pos}{\sum }}\overset{N}{\underset{m\in \left\{cx,cy,w,h\right\}}{\sum }}x_{ij}^{k}smooth_{L1}\left(l_{i}^{m}-{\hat{g}}_{j}^{m}\right)$$

(3) $${\hat{g}}_{j}^{cx}=(g_{j}^{cx}-d_{i}^{cx})/d_{i}^{w}\;{\hat{g}}_{j}^{cy}=\left(g_{j}^{cy}-d_{i}^{cy}\right)/d_{i}^{h}\;{\hat{g}}_{j}^{w}=\log\left(g_{j}^{w}/d_{i}^{w}\right)\;{\hat{g}}_{j}^{h}=\log\left(g_{j}^{h}/d_{i}^{h}\right)$$

(4) $$smooth_{L1}\left(x\right)=\{\begin{array}{c} 0.5x^{2}\;\;if\left|x\right|<1\\ \left|x\right|-0.5\;\;otherwise \end{array}$$

The localization loss (see Equation (2)) uses the $smooth_{L1}$ loss as the predicted box (l) and ground truth box (g) parameters. $x_{ij}^{k}$ is a value of 0 or 1, and $x_{ij}^{k}$ indicates whether the i-th bounding box with coordinates ($l_{i}^{cx},l_{i}^{cy},l_{i}^{w},l_{i}^{y})$ is matched to the j-th ground truth box with coordinates ($g_{j}^{cx},g_{j}^{cy},g_{j}^{w},g_{j}^{y})$ for any object.

(5) $$L_{conf}\left(x,c\right)=-\overset{N}{\underset{i\in Pos}{\sum }}x_{ij}^{p}\log\left({\hat{c}}_{i}^{p}\right)-\underset{i\in Neg}{\sum }\log\left({\hat{c}}_{i}^{0}\right)$$

The confidence loss (see Equation (5)) is a softmax function for multiple classes of confidence (c), where ${\hat{c}}_{i}^{p}=\exp\left(c_{i}^{p}\right)/\underset{p}{\sum }\exp\left(c_{i}^{p}\right)$. $x_{ij}^{p}$ is a value of 0 or 1; if $x_{ij}^{p}$ = 1, it means that it matches the i-th default bbox and the j-th ground truth bbox of the p category. We used the Adam algorithm to optimize the loss function to find the optimal solution. We used a learning rate of $10^{-3}$, with a decay of 0.0, epsilon of $10^{-8}$. That is, Pos :iou>0.5 and Neg :iou ≤0.5. The model was trained for up to 200 epochs using the early stopping technique to prevent overfitting. As a backbone network can use multiple architectures, in this study, we utilized modified VGGNet, ResNet, and DenseNet architectures. The detailed network structures and the total number of weight parameters are listed in Table 3, Table 4 and Table 5.

## 4. Experimental Results

In this experiment, the SSD model was used to classify 11 parrot species, and the performances of various backbone networks were analyzed as shown in Table 3, Table 4 and Table 5. After data collection and preprocessing, as described in Section 3.1, the dataset was used for the experiment. The dataset comprised 11 parrot species, including images taken indoors and outdoors in various environments.

**Figure 4:**
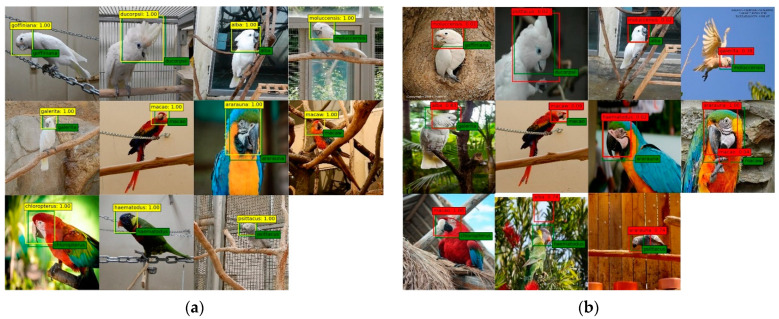
Examples of parrot species classification results (green: ground-truth, yellow: correct prediction, red: incorrect prediction). (**a**) Examples of correctly detected and classified. (**b**) Examples of incorrectly detected or classified.

Figure 4 shows the correct and incorrect classification results for the 11 parrot species. Misclassification results include cases where a background other than the parrot’s head area was detected due to the ambiguity between the background and the head area.

The metric used to evaluate the performance of the object detection model was mAP [31], which is defined as the average value of the AP value for each class. The AP values were calculated from the precision-recall curve, which can be obtained by the precision (see Equation (6)) and recall (see Equation (7)) measures, where TP, FP, and FN represent true positives, false positives, and false negatives, respectively, as defined in Table 6. An intersection of union (IoU) value of 0.5 [29,35] or greater between the predicted and ground truth bbox is considered a TP.

(6) $$\mathrm{Precision}=\frac{\mathrm{TP}}{\mathrm{TP}+\mathrm{FP}}=\frac{\mathrm{TP}}{\mathrm{All}\;\mathrm{detection}}$$

(7) $$\mathrm{Recall}=\frac{\mathrm{TP}}{\mathrm{TP}+\mathrm{FN}}=\frac{\mathrm{TP}}{\mathrm{All}\;\mathrm{ground}\;\mathrm{truth}}$$

In addition, when using the RTX 2080 with Max-Q design, we measured the model inference time as the average value for 10 images. Table 7 lists the performance of the proposed models and the model inference time.

As shown in Table 7, the inference time for each model is less than 1 s. In this study, because it is important to accurately classify endangered species, we used the model performance as the basis for selection. The DenseNet18 model performed the best, while the DenseNet50 model performed the worst. In particular, ResNet and DenseNet, which reuse feature information in the previous layer, performed better than VGG16, which uses a plain CNN structure. This is a structural feature of plain CNNs; thus, it can be seen that the feature information from the previous layer was not completely transferred to the next layer.

**Figure 5:**
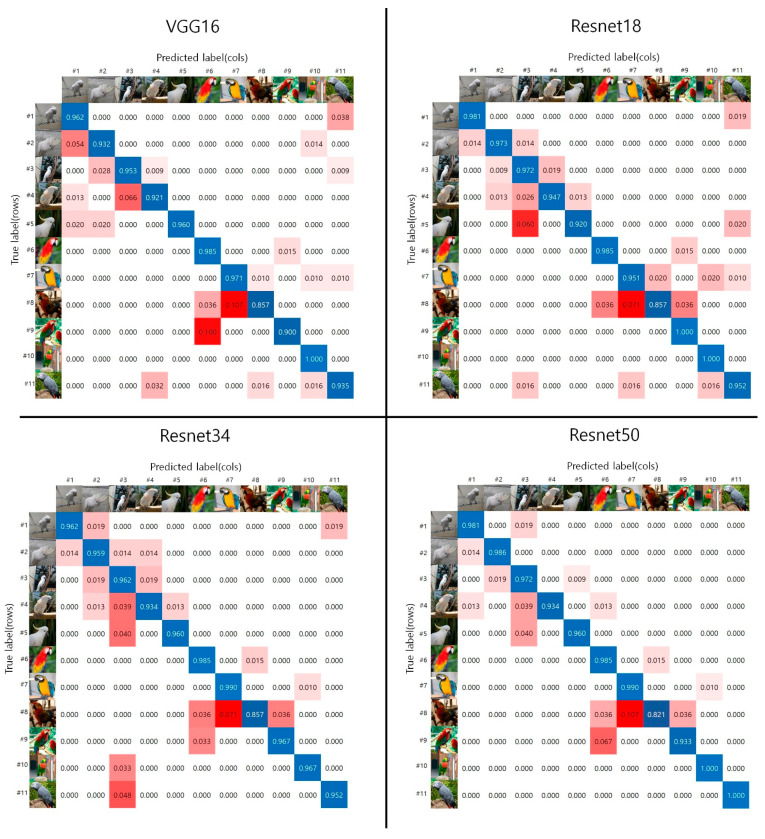

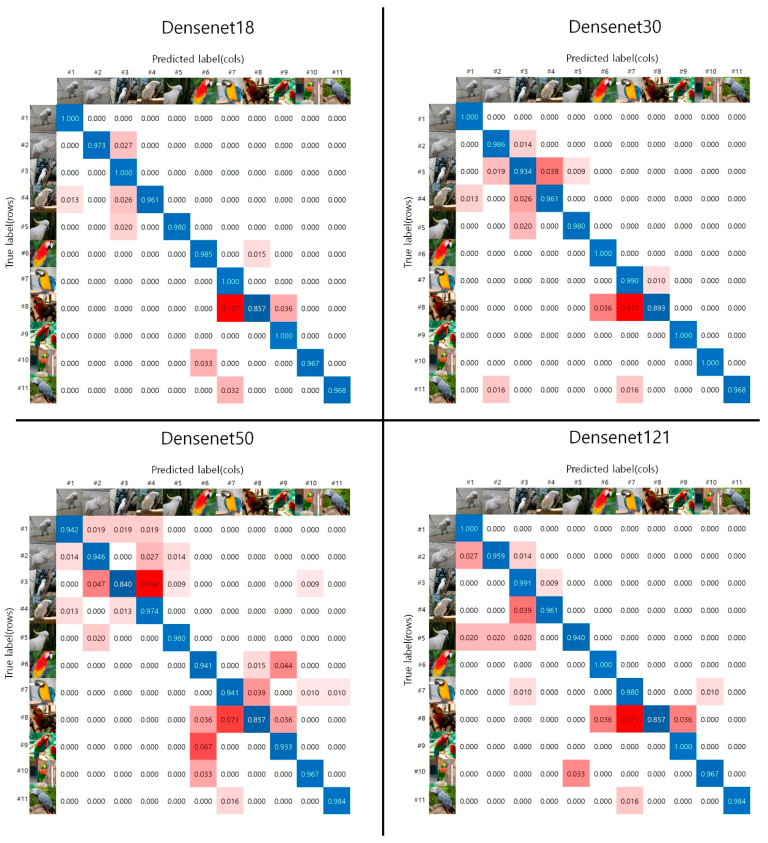
The confusion matrices of the 11 parrot species for each model proposed. The on-diagonal results (blue tones) are the correct predictions, whereas the red tones are the incorrect predictions.

A confusion matrix is provided to help understand the performance of each network model proposed in this study (see Figure 5). Normalization is used to make the values in a dataset between 0 and 1; with values that are closer to 1 indicating a correct prediction. The *x*-axis is the predicted label, and the *y*-axis is the correct answer label. The blue tone on the diagonal is the correct prediction result, and the red and yellow tones are the incorrect prediction results. As shown by the DenseNet18 confusion matrix, it has the lowest number of error cases and the best performance, compared to the other models.

## 5. Discussion

In this study, we used the SSD model to compare the classification performance of different CNN structures (i.e., VGGNet, ResNet, and DenseNet) on 11 parrot species designated as endangered by CITES. To make a fair comparison, the final optimal parameters learned for each architecture might be different, but the convolutional layer hyperparameters were set equal to ‘He initialization’ [36]. These results are valid only in environments with the same hyperparameters. To make a model suitable for parrot classification, each architecture was trained without using pre-training. Among the parrots designated as endangered by CITES, we selected 11 species with similar and frequent illegal customs cases.

As shown in Figure 5, Species #1 to #5 presented in the confusion matrix belong to the Cacatua genus. These species have mainly white feathers with yellow wings and tail and black beak. Species #6 to #9 belong to the Ara genus. These species have characteristic facial patches around their eyes and huge beaks that bend down. The biological characteristics of these Cacatua and Ara genera are clearly different for each parrot with respect to its beak shape, crest, and face patch. Therefore, we used the head area to classify the 11 parrot species.

**Figure 6:**
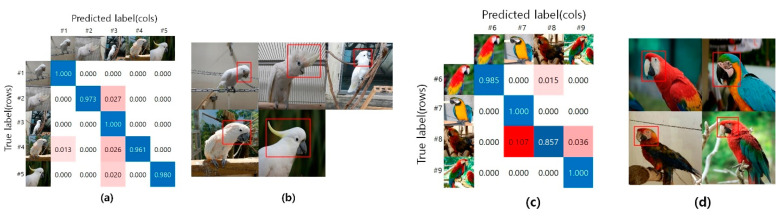
The sample data used for deep neural network from #1 to #9. (**a**) DenseNet18 model confusion matrix corresponding to #1 to #5; (**b**) Cacatua genus; (**c**) DenseNet18 model confusion matrix corresponding to #6 to #9; (**d**) Ara genus.

In general, as seen with the naked human eye, the Ara genus, which has various colors, seems to be classified better than the Cacatua genus, which is a white-colored species. However, as shown by the experimental results using each deep learning model in Figure 5, the white-colored species belonging to the Cacatua genus had a better classification result. As shown in Figure 6, species belonging to the Cacatua genus have similar colors and different external shapes, whereas those belonging to the Ara genus have various colors and similar external shapes. Therefore, it can be said that the features used in deep learning model training are learned relatively well for appearance features rather than color features. Additionally, using artificial intelligence techniques, it is possible to classify the Cacatua and Ara genera, which are difficult to discriminate with the naked eye; thus, it may be helpful for non-professionals as well.

## 6. Conclusions

In this paper, we proposed a deep learning-based object detection model to classify 11 parrot species that are designated as endangered by CITES. Among the CNN architectures (i.e., VGGNet, ResNet, and DenseNet), the DenseNet18 model showed the best performance. We also used data augmentation techniques to address data shortages and to reduce the risk of overfitting. As a result of the experiment, we found the appearance features to be relatively better than the color features in the model learning stage for classifying the Cacatua and Ara genera. Additionally, we found the ResNet and DenseNet architectures, which reuse previous layer information, to have better performance compared to VGGNet, which has a plain CNN structure.

It is important to reduce the false negative rate because the aim is to classify the endangered species. In this study, the false negative rate and false positive rate were derived using Bayesian decision theory [37]. However, the false negative rate can be reduced by adjusting the confidence threshold, even if the false positive rate is increased, which can be managed as per the need.

These methods can increase the efficiency of research on endangered species. Further, the proposed model for customs clearance systems installed at airports and ports can help nonexperts with inspecting specific animal species by saving money and time as well as simplifying the process. In future work, we plan to improve classification performance for species belonging to the Cacatua genus by adding other Cacatua species other than the species used in this study. In deep learning, performance improves as the size of data increases, and therefore this study contributes to performance improvement by creating a large database using additional data collection and data augmentation at Seoul Grand Park Zoo. To augment insufficient data, we plan to use a 3D shape-based data augmentation method that considers not only the geometric transformation of the 2D image but also the posture and capturing angle of the animal. Furthermore, by adopting and comparing the state-of-the-art CNN models, we will derive the optimal model suitable for the classification of a specific animal species. Moreover, we will analyze the biological properties and study how to add features, such as the torso and feet, to improve performance.

## Figures and Tables

**Table 1:** Dataset of the 11 parrot species after data augmentation.

Scientific Name	Train Set	Validation Set	Test Set
*Cacatua goffiniana*	3248	50	52
*Cacatua ducorpsii*	3300	70	74
*Cacatua alba*	2880	102	106
*Cacatua moluccensis*	3460	74	76
*Cacatua galerita*	3136	48	50
*Ara macao*	3080	66	68
*Ara ararauna*	2808	100	102
*Camelot macaw*	3224	26	28
*Ara chloroptera*	3224	26	30
*Trichoglossus Haematodus*	3380	28	30
*Psittacus erithacus*	2740	58	62
Total	34,480	648	678

**Table 2:** Overview of our dataset of the 11 parrot species designated as endangered by CITES.

**Picture**	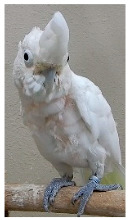	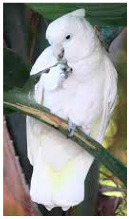	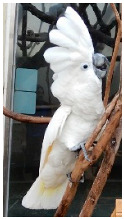	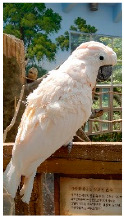
Common name	Goffin’s cockatoo	Solomons cockatoo	The white cockatoo	Moluccan cockatoo
Scientific name	Cacatua goffiniana	Cacatua ducorpsii	Cacatua alba	Cacatua moluccensis
CITES listing	Appendix I	Appendix II	Appendix II	Appendix I
**Picture**	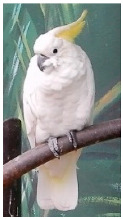	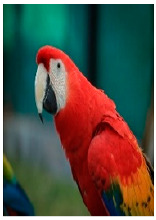	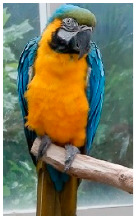	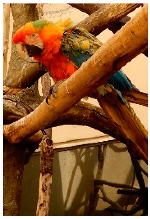
Common name	The sulphur-crested cockatoo	Red and yellow macaw	Blue and gold macaw	Camelot macaw
Scientific name	Cacatua galerita	Ara macao	Ara ararauna	(Ara ararauna × Ara macao) × Ara cholroptera
CITES listing	Appendix II	Appendix I	Appendix II	
**Picture**	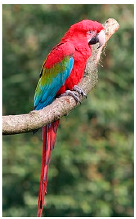	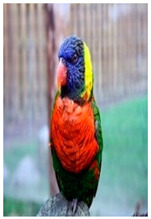	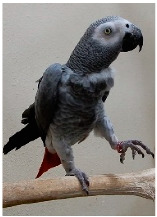	
Common name	Red and green macaw	Rainbow lorikeet	Gray parrot	
Scientific name	Ara chloroptera	Trichoglossus haematodus	Psittacus erithacus	
CITES listing	Appendix II	Appendix II	Appendix I	

**Table 3:** Model specification of VGGNet based on SSD architecture.

Layers	Output Size (Width× Height×Channel)	Specification
Conv × 2	300 × 300 × 64	3 × 3 Conv, stride 1, name: conv1_1
	300 × 300 × 64	3 × 3 Conv, stride 1, name: conv1_2
Pooling	150 × 150 × 64	2 × 2 Max Pool, stride 2
Conv × 2	150 × 150 × 128	3 × 3 Conv, stride 1, name: conv2_1
	150 × 150 × 128	3 × 3 Conv, stride 1, name: conv2_2
Pooling	75 × 75 × 128	2 × 2 Max Pool, stride 2
Conv × 3	75 × 75 × 256	3 × 3 Conv, stride 1, name: conv3_1
	75 × 75 × 256	3 × 3 Conv, stride 1, name: conv3_2
	75 × 75 × 256	3 × 3 Conv, stride 1, name: conv3_3
Pooling	38 × 38 × 256	2 × 2 Max Pool, stride 2
Conv × 3	38 × 38 × 512	3 × 3 Conv, stride 1, name: conv4_1
	38 × 38 × 512	3 × 3 Conv, stride 1, name: conv4_2
	38 × 38 × 512	3 × 3 Conv, stride 1, name: conv4_3
Pooling	19 × 19 × 512	2 × 2 Max Pool, stride 2
Conv × 3	19 × 19 × 512	3 × 3 Conv, stride 1, name: conv5_1
	19 × 19 × 512	3 × 3 Conv, stride 1, name: conv5_2
	19 × 19 × 512	3 × 3 Conv, stride 1, name: conv5_3
Pooling	19 × 19 × 512	3 × 3 Max Pool, stride 1
Weight parameters (unit: million)	25	

Note that each “Conv” layer shown in the table corresponds to the composite function sequence Conv-ReLU.

**Table 4:** Model specification of ResNet based on SSD architecture.

**Layers**	Output Size (Width×Height×Channel)	18-Layer	34-Layer	50-Layer
Conv	75 × 75 × 64	7 × 7 Conv, stride 2 3 × 3 Max Pool, stride 2		
Block (1)	75 × 75 × 64	$\left[\begin{array}{c} 3\times 3\;\mathrm{Conv}\\ 3\times 3\;\mathrm{Conv} \end{array}\right]\times 2$	$\left[\begin{array}{c} 3\times 3\;\mathrm{Conv}\\ 3\times 3\;\mathrm{Conv} \end{array}\right]\times 3$	$\left[\begin{array}{c} 1\times 1\;\mathrm{Conv}\\ 3\times 3\;\mathrm{Conv}\\ 1\times 1\;\mathrm{Conv} \end{array}\right]\times 3$
Block (2)	38 × 38 × 128	$\left[\begin{array}{c} 3\times 3\;\mathrm{Conv}\\ 3\times 3\;\mathrm{Conv} \end{array}\right]\times 2$	$\left[\begin{array}{c} 3\times 3\;\mathrm{Conv}\\ 3\times 3\;\mathrm{Conv} \end{array}\right]\times 4$	$\left[\begin{array}{c} 1\times 1\;\mathrm{Conv}\\ 3\times 3\;\mathrm{Conv}\\ 1\times 1\;\mathrm{Conv} \end{array}\right]\times 4$
Block (3)	19 × 19 × 256	$\left[\begin{array}{c} 3\times 3\;\mathrm{Conv}\\ 3\times 3\;\mathrm{Conv} \end{array}\right]\times 2$	$\left[\begin{array}{c} 3\times 3\;\mathrm{Conv}\\ 3\times 3\;\mathrm{Conv} \end{array}\right]\times 6$	$\left[\begin{array}{c} 1\times 1\;\mathrm{Conv}\\ 3\times 3\;\mathrm{Conv}\\ 1\times 1\;\mathrm{Conv} \end{array}\right]\times 6$
Weight parameters (unit: million)		10	16	23

Note that each “Conv” layer shown in the table corresponds to the composite function sequence BN-ReLU-Conv.

**Table 5:** Model specification of DenseNet based on SSD architecture.

**Layers**	Output Size (Width×Height×Channel)	18-Layer	30-Layer	50-Layer	121-Layer
Conv	75 × 75 × 64	7 × 7 Conv, stride 2 3 × 3 max pooling, stride 2			
Dense block (1)	75 × 75 × 256	$\left[\begin{array}{c} 1\times 1\;\mathrm{Conv}\\ 3\times 3\;\mathrm{Conv} \end{array}\right]\times 6$	$\left[\begin{array}{c} 1\times 1\;\mathrm{Conv}\\ 3\times 3\;\mathrm{Conv} \end{array}\right]\times 6$	$\left[\begin{array}{c} 1\times 1\;\mathrm{Conv}\\ 3\times 3\;\mathrm{Conv} \end{array}\right]\times 8$	$\left[\begin{array}{c} 1\times 1\;\mathrm{Conv}\\ 3\times 3\;\mathrm{Conv} \end{array}\right]\times 6$
Transition (1)	75 × 75 × 256	1 × 1 Conv			
	38 × 38 × 256	2 × 2 average pooling, stride 2			
Dense block (2)	38 × 38 × 384	$\left[\begin{array}{c} 1\times 1\;\mathrm{Conv}\\ 3\times 3\;\mathrm{Conv} \end{array}\right]\times 4$	$\left[\begin{array}{c} 1\times 1\;\mathrm{Conv}\\ 3\times 3\;\mathrm{Conv} \end{array}\right]\times 8$	$\left[\begin{array}{c} 1\times 1\;\mathrm{Conv}\\ 3\times 3\;\mathrm{Conv} \end{array}\right]\times 10$	$\left[\begin{array}{c} 1\times 1\;\mathrm{Conv}\\ 3\times 3\;\mathrm{Conv} \end{array}\right]\times 12$
Transition (2)	38 × 38 × 384	1 × 1 Conv			
	19 × 19 × 384	2 × 2 average pooling, stride 2			
Dense block (3)	19 × 19 × 512	$\left[\begin{array}{c} 1\times 1\;\mathrm{Conv}\\ 3\times 3\;\mathrm{Conv} \end{array}\right]\times 4$	$\left[\begin{array}{c} 1\times 1\;\mathrm{Conv}\\ 3\times 3\;\mathrm{Conv} \end{array}\right]\times 8$	$\left[\begin{array}{c} 1\times 1\;\mathrm{Conv}\\ 3\times 3\;\mathrm{Conv} \end{array}\right]\times 13$	$\left[\begin{array}{c} 1\times 1\;\mathrm{Conv}\\ 3\times 3\;\mathrm{Conv} \end{array}\right]\times 24$
Transition (3) without pooling	19 × 19 × 512	1 × 1 Conv			
Dense block (4)	19 × 19 × 640	$\left[\begin{array}{c} 1\times 1\;\mathrm{Conv}\\ 3\times 3\;\mathrm{Conv} \end{array}\right]\times 4$	$\left[\begin{array}{c} 1\times 1\;\mathrm{Conv}\\ 3\times 3\;\mathrm{Conv} \end{array}\right]\times 8$	$\left[\begin{array}{c} 1\times 1\;\mathrm{Conv}\\ 3\times 3\;\mathrm{Conv} \end{array}\right]\times 19$	$\left[\begin{array}{c} 1\times 1\;\mathrm{Conv}\\ 3\times 3\;\mathrm{Conv} \end{array}\right]\times 16$
Transition (4) without pooling	19 × 19 × 640	1 × 1 Conv			
Weight parameters (unit: million)		13	20	32	39

The growth rate K = 32 was used for each dense block. Note that each “Conv” layer shown in the table corresponds to the composite function sequence BN-ReLU-Conv.

**Table 6:** Confusion matrix.

		Predicted Class	
		Positive	Negative
**Actual class**	Positive	TP	FN
	Negative	FP	TN

**Table 7:** Performance for the model.

Network Model	Mean Average Precision (Unit: %)	Inference Time (Unit: s)
VGG 16	95.7	0.29
ResNet18	96.4	0.25
ResNet34	96	0.28
ResNet50	96	0.33
DenseNet18	96.6	0.38
DenseNet30	96.3	0.47
DenseNet50	95.6	0.64
DenseNet121	96.2	0.69

## Data Availability

Upon request by e-mail to the corresponding author, it may be provided after review depending on the purpose of use.
